# Prevalence of Intestinal Parasitic Infection among HIV Positive Persons Who Are Naive and on Antiretroviral Treatment in Hiwot Fana Specialized University Hospital, Eastern Ethiopia

**DOI:** 10.1155/2013/324329

**Published:** 2013-06-11

**Authors:** Zelalem Teklemariam, Degu Abate, Habtamu Mitiku, Yadeta Dessie

**Affiliations:** ^1^Department of Medical Laboratory Sciences, College of Health and Medical Sciences, Haramaya University, P.O. Box 235, Harar, Ethiopia; ^2^Department of Public Health, College of Health and Medical Sciences, Haramaya University, P.O. Box 235, Harar, Ethiopia

## Abstract

*Background*. Intestinal parasitic infection affects the health and quality of life of people living with HIV. This study was aimed to determine the prevalence of intestinal parasites among HIV positive individuals who are naive and who are on antiretroviral treatment (ART) in Hiwot Fana Specialized University Hospital, Eastern Ethiopia. *Methods*. A comparative cross-sectional study was conducted on 371 (112 ART-naive group and 259 on ART) HIV positive individuals. Stool specimens were collected and examined by direct wet mount, formol ether concentration technique, and modified ziehl-Neelsen methods. *Results*. The overall prevalence of intestinal parasitic infections was 33.7%; it was significantly higher among the study participants who were ART-naive group (45.5%) (AOR: 2.60(1.56,4.34)) and diarrheic (53.3%) (AOR: 2.30(1.34,3.96)) and with CD_4_ count <200 cells/**μ**L (46%) (AOR: 2.07(1.06,4.04)). The most commonly identified parasites were *Entamoeba histolytica/E. dispar *(13.5%), *Giardia lamblia* (8.1%), *Strongyloides stercoralis* (4.0%), and *Cryptosporidium* species (2.2%). *Conclusion*. HIV positive individuals with diarrhea and low CD_4_ count and ART naive groups were more infected with intestinal parasites than their counterparts. Early stool examination and treatment of intestinal parasites for HIV/AIDS patients is essential.

## 1. Introduction

Globally about 3.5 billion people are infected with intestinal parasite. Out of whom, 450 million are suffering from its illness [1, 2]. The prevalence of intestinal parasitic infection is high in Sub-Saharan Africa, where the majority of HIV/AIDS cases are from [2, 3]. Parasites are common infections among HIV/AIDS patients [4–7]. Diarrhea causing opportunistic parasites like *Cryptosporidium parvum *and *Isospora belli* is common among HIV positive persons with CD_4_ count less than 200 cells/*μ*L [4, 5]. 

Antiretroviral treatment (ART) increases the length and quality of life and productivity of patients by improving survival and decreasing the incidence of opportunistic infections in people with HIV through reduction of the viral load and increasing the level of CD_4_ cells [8]. Nevertheless, in Ethiopia, few studies have tried to investigate the extent of intestinal parasitic infections in relation to ART experiences and CD4 count [4, 5]. 

Therefore, we investigated the prevalence of intestinal parasites among HIV positive persons who were naive and who were on ART in Hiwot Fana Specialized University Hospital, Eastern Ethiopia.

## 2. Material and Methods 

### 2.1. Study Setting

 The study was conducted in Hiwot Fana Specialized University Hospital, Harar, Ethiopia. It is a teaching hospital of Haramaya University. The hospital is found in Harari National Regional State which is one of the Federal Democratic Republic of Ethiopia regional states located 515 km from Addis Ababa. In Harar, there are six hospitals and eight health centers. The health service coverage of the region was estimated to be above 100%. A comparative cross-sectional study was conducted among Naive and on ART HIV positive persons in Hiwot Fana Specialized University Hospital from March to April, 2011. 

### 2.2. Study Participant

The study population was all the HIV positive individuals who were on ART and pre ART care in the ART unit of the hospital during the study period. Individuals who were on parasitic treatments during the data collection period were excluded. The prevalence of intestinal parasites among ART-naive group HIV positive individuals was taken as 50% and to detect a difference of 15% between the two groups with the assumption of 95% confidence level (CL), power of 80% (0.84) and ratio 2 : 1 of those on ART and ART-naive group. Double proportion formula was applied to calculate the sample size which was 274 on ART and 137 ART-naive group. A 5% nonresponse rate was added and the final calculated sample size was 431. Study participants were selected by simple random sampling techniques.

### 2.3. Data Collection and Laboratory Investigation Procedures

#### 2.3.1. Stool Collection and Examination

Small pieces of labeled clean plastic sheets and wooden applicator sticks were distributed and the participants were instructed to bring sizable stool specimen of their own. With the provision of specimen, each participant was interviewed for sociodemographic variables. Each stool specimen was initially assessed for consistency. Then, it was examined by direct wet mount method using normal saline (0.85% NaCl solution) at Hiwot Fana Specialized University Hospital Laboratory in order to prevent the loss of motile stage of parasites. Lugol's iodine was used to detect the cyst of intestinal parasites. The remaining sample was preserved with 10% formalin and transported to the laboratory of Medical Laboratory Sciences Department Haramaya University. In the laboratory, it was examined by formol ether concentration technique and modified Zeihl-Neelsen method (for detection of opportunistic parasites—*Cryptosporidium* species, *Cyclospora cayetanensis,* and *Isospora belli*) [9]. The most recent CD_4_ T-cells counts of the participants were obtained from their ART fellow-up record in the hospital.

### 2.4. Data Processing and Analysis

Data were entered into Epideta Version 3.1 and transported to SPSS Version 16 software for analysis. The prevalence of intestinal parasites was determined in relation to different variables Pearson's chi square test was used to assess statistical significance difference between proportions. Bivariate and multivariate analyses were performed to assess crude and adjusted ratio. A statistical test result was reported as significant when its *P* value was less than 0.05.

### 2.5. Ethical Consideration

Ethical clearance was obtained from Haramaya University Colleges of Health and Medical Sciences Institutional Research and Ethical Review Committee. All the participants were explained about the purpose and their right to participate or not to participate in this study. Those who gave their written consent participated in this study. Moreover, all personal information of the participants was kept confidential. Those participants who were found positive for intestinal parasitic infection were treated free of charge using the standard drugs by nurses in ART unit. ART follow-up record was also retrieved by nurses working in ART unit.

## 3. Results

### 3.1. Sociodemographic Characteristics of Study Participants

A total of 259 on ART and 112 ART-naive group HIV positive clients participated in the study, and the response rate was 86.7%. The mean age of the participants was 33.6 (SD ±10.04) and many of the ART-naive group (68.5%) and on ART (70%) participants were female. The majority of the study subjects were in the age group of 25–34, married, daily laborers, and urban dwellers (Table 1). 

### 3.2. The Distribution of Parasite Species

The overall prevalence of intestinal parasites among the study participants was 33.7% (125/371). It was significantly higher in the ART-naive group (45.5% (51/112)) compared to those on ART (28.6% (74/259)) (*P* = 0.002). Some were infected with nonopportunistic (31.8%) and very few with opportunistic (3.5%) parasites. 

Eleven parasite species were detected: *Entamoeba histolytica/Entamoeba dispar *(13.4% on ART-naive group and 13.5% on ART group (*P* > 0.05)) and *Giardia lamblia* (7.1% on ART-naive group and 8.5% on ART group) were the common ones. Opportunistic parasites like Crypotospriudum (7.1%) and *Isospora belli* (4.5%) were found only in ART-naive group group. 

Some of the ART-naive group (26.8%) and few of the on ART (17.4%) subjects were diarrheic (*P* < 0.05); most of them were infected with *G. lamblia* (*P* > 0.05), Crypotospriudum species, *I. belli, and S. Stercolaris.* The overall prevalence of intestinal parasites was significantly higher among diarrheic (53.3% (40/75)) as compared to nondiarrheic (28.7% (85/296)) study participants (*P* < 0.05) (Table 2).

About 31% (31/100), 17.3% (34/196), and 13.3% (10/75) of the study participants with CD_4_ count less than 200 cells/*μ*L, 200–499 cells/*μ*L and greater than or equal to 500 cells/*μ*L, respectively, were diarrheic. The prevalence of diarrhea is significant in participants with CD_4_ count less than 200 cells/*μ*L (*P* = 0.005). 

In the bivariate analysis, age, sex, occupation, residence, education, and marital status of the participants did not show significant association with prevalence of intestinal parasites. Participants with CD_4_ count less than 200 cells/*μ*L (COR: 1.93; CI: 1.03, 3.61), diarrheic (COR: 2.84; CI: 1.69, 4.77), and ART-naive group (COR: 2.09; CI: 1.32, 3.31) had higher risk of intestinal parasites. The participants with CD_4_ count <200 cells/*μ*L were 2.07 times more likely to be infected with intestinal parasites than those with CD_4_ count ≥500 cells/*μ*L. The diarrheic participants and the ART-naive group ones were 2.30 and 2.60 times more likely to be infected with intestinal parasites (Table 3).

## 4. Discussions

Intestinal parasitic infections are the major causes of morbidity and mortality in HIV positive patients [4–7, 10]. In this study, about 33.7% of the participants were infected with intestinal parasites. The result is almost similar to the ones reported from Afar, Ethiopia [5], Cameroon [11], and Saudi Arabia [12], but lower than those from Jimma [13], Hawassa [4], Bahir Dar [14], Dire Dawa and Afar [5] of Ethiopia, India [15], Kenya [16], and Jakarta [17]. But it is higher than a finding in Senegal [18]. The difference in the prevalence might be due to differences in geographical location, sensitivity of diagnostic techniques, study participants' immunity status, environmental hygiene, socioeconomic status, access to safe water supply, or other. 

Several species of protozoa and other intestinal parasites have been associated with acute and chronic diarrhea and even weight loss in HIV/AIDS patients [19, 20]. In this study, *Entamoeba histolytica/E. dispar* and *Giardia lamblia* were the commonest nonopportunistic protozoa. The overall prevalence of *Entamoeba histolytica/E. dispar *was 13.5%. The finding is similar with 13% report in Ethiopia [5]. But it is higher than a report in Ethiopia (4.2%) [14], Saudi Arabia (5.2%) [12], and Jakarta (0.3%) [17]. It is lower than the one in Kenya (58.3%) [16] and Ethiopia (23.8%) [13]. The overall prevalence of *Gardia lamblia* was 8.1%. It is slightly lower than 10.6% in Ethiopia [13]. However, it is higher than report of 1.1% in Ethiopia [14], 0.6% in Senegal, and 1.9% in Jakarta [17]. It is also lower than report of 16.6% in Kenya [16] and 16% in Ethiopia [5].

Opportunistic protozoa like Crypotospriudum species and *Isospora belli *were also identified in this study. The prevalence of Crypotospriudum species was 2.2% and this is lower than the findings from other studies which are found in the range of 4.9% to 15.8% [5, 12–15, 17]. While *Isospora belli* was found at 1.3%. This is lower than other studies which are found in the range of 3.9% to 11.7% [5, 13–15]. The lower prevalence of both parasites in this study might be due to that our study participants are in the ART care who were taking ART and/or treatment for opportunistic infection. The other reason might be due to difference in immunity, diarrheic status, environmental and personal hygiene of the study participants. 

Other nonopportunistic intestinal helimiths were identified at the rate of ranging from 0.5% to 4.0%. The highest prevalent helminth was *Strongyloides stercoralis*. This is similar to study carried out in Ethiopia [13], but it is slightly lower than another study in Ethiopia [14] and Saudi Arabia [12]. The effect of *Strongyloides stercoralis* in HIV infected patients, which is disseminated strongloidiasis, was reported in another study [21]. 

In this study, we tried to compare the prevalence of intestinal parasites with the diarrheic status, CD_4_ count, and ART experience of HIV positive persons. The prevalence of intestinal parasites was significantly higher among diarrheic as compared to nondiarrheic groups. Similar reports are found in other studies [12, 15, 16]. The most common diarrheal causing parasites were *E. histolytica, Gardia lamblia*, Crypotospriudum species, *Isospora belli,* and *Strongyloides stercoralis*. Similarly, there are reports which showed that diarrhea can be caused by opportunistic and nonopportunistic parasites [5, 15, 22].

 The association between opportunistic parasitic infection and HIV is widely reported [4, 15, 23]. However, in this study, most of the parasites identified were nonopportunistic. The relationship between nonopportunistic parasite and HIV was not well established. Even though the defense against them might be damaged by HIV, the exposure to this parasites are likely to occur independent of HIV infection but heavier parasitic load might accumulate as well as experience of delayed clearance of parasite in individual with concurrent HIV induced immunosupperssion [23, 24].

The prevalence of intestinal parasites was highly significant among those study participants with CD_4_ count <200 cells/*μ*L in this study. This is consistent with other studies [4, 5, 15]. The correlation of CD_4_ count with opportunistic parasites could not be assessed because of small number of individuals infected with Crypotospriudum species and *Isospora belli*. However, the association of these two parasites with HIV positive persons, who have CD_4_ count <200 cells/*μ*L, is reported in other studies [4, 5, 15, 23]

The prevalence of intestinal parasites was significantly higher when ART-naive group compared to those on ART study participants. Opportunistic parasites, which are Crypotospriudum species and *Isospora belli*, were found only in ART-naive group group. This is similar to study conducted in Ethiopia [5]. ART increases the immunity status HIV positive persons and decreases the incidence of opportunistic infections [8]. 

The study was not without limitations. The detection method for *Microsoporidia* was not employed and sensitive diagnostic techniques for *Strongyloides stercoralis* and *Enetrobius vermicularis* were not used. Therefore, the prevalence intestinal parasites in our study participant may have been underestimated. HIV negative control groups were not included in this study which was the other limitation.

## 5. Conclusions 

The prevalence of intestinal parasite was high. Opportunistic and nonopportunistic parasites were identified with a different rate. The prevalence of intestinal parasites was higher among those HIV infected individuals with diarrhea, low CD_4_ count, and ART-naive group groups. Those results posit the need for considering early detection and treatment of intestinal parasites in HIV infected individuals in order to reduce their morbidity. This seeks great attention by those clinical service providers who are working in the ART unit. Adherence counseling of ART, health information dissemination on environmental, and personal hygiene should also be given to HIV/AIDS patients. In addition further large scale study by using different diagnostic techniques, HIV negative control and assessing predisposing factors of intestinal parasites is recommended.

## Figures and Tables

**Table 1:** Sociodemographic characteristics of naïve ART and on ART HIV positive study participants at Hiwot Fana Specialized University Hospital, Harar, Eastern Ethiopia, 2011.

Variables	Naïve ART	On ART	Total
	*N* (%)	*N* (%)	*N* (%)
Age (in year)			
15–24	35 (31.5%)	40 (15.6%)	75 (20.4)
25–34	51 (45.9%)	121 (47.3%)	172 (46.9)
≥35	25 (22.5%)	95 (37.1%)	120 (32.7)
Sex			
Male	35 (31.5)	76 (29.3)	111 (30)
Female	76 (68.5)	183 (70.7)	259 (70)
Educational status			
Illiterate	27 (24.1)	56 (21.6)	83 (22.4)
Read and write	3 (2.7)	7 (2.7)	10 (2.7)
Elementary school	49 (43.8)	94 (36.3)	143 (38.5)
High school and above	33 (29.5)	102 (39.4)	135 (36.4)
Marital status			
Single	30 (27.5)	56 (21.6)	86 (23.4)
Married	55 (50.5)	112 (43.2)	167 (45.4)
Divorced	14 (12.8)	55 (21.2)	69 (18.8)
Widowed	10 (9.2)	36 (13.9)	46 (12.5)
Occupation			
Government employee	14 (12.8)	56 (21.8)	70 (19.1)
Nongovernmental organization	5 (4.6)	13 (5.1)	18 (4.9)
Farmer	2 (1.8)	11 (4.3)	13 (3.6)
Merchants	23 (21.1)	47 (18.3)	70 (19.1)
Daily laborer	27 (24.8)	66 (25.7)	93 (25.4)
House wife	19 (17.4)	26 (10.1)	45 (12.3)
Students	1 (0.9)	3 (1.2)	4 (1.1)
Other	12 (11.0)	16 (6.2)	28 (7.7)
No job	6 (5.5)	19 (7.4)	25 (6.8)
Residence			
Rural	23 (21.3)	65 (25.3)	88 (24.1)
Urban	85 (78.7)	192 (74.2)	277 (75.9)

**Table 2:** Intestinal parasite species identified with ART and diarrheic status of HIV positive study participants at Hiwot Fana Specialized University Hospital, Harar, Eastern Ethiopia, 2011.

Parasite species	Naïve ART (*N* = 112) Number of pos. (%)	On ART (*N* = 259) Number of pos. (%)	Total (*N* = 371) Number of pos. (%)	*P* value	Diarrheic (*n* = 75) Number of pos. (%)	Nondiarrheic (*n* = 296) Number of pos. (%)	*P* value
*Entamoeba histolytica/E. dispar *	15 (13.4)	35 (13.5)	50 (13.5)	0.975	10 (13.3)	40 (13.5)	0.967
* Giardia lamblia *	8 (7.1)	22 (8.5)	30 (8.1)	0.661	9 (12)	21 (7.1)	0.164
*Strongyloides stercoralis*	6 (5.4)	9 (3.5)	15 (4.0)	—	9 (12)	6 (2)	—
*Crypotospriudum* species	8 (7.1)	0 (0)	8 (2.2)		7 (9.3)	1 (0.3)	—
Hookworm	3 (2.7)	4 (1.5)	7 (1.9)	—	0 (0)	7 (2.4)	—
* Isospora belli *	5 (4.5)	0 (0)	5 (1.3)	—	5 (6.7)	0 (0)	—
* Hymenolepis nana *	1 (0.9)	4 (1.5)	5 (1.3)	—	0 (0)	5 (1.7)	—
* Taenia saginata *	0 (0)	3 (1.2)	3 (0.8)	—	0	3 (1.0)	—
*Ascaris lumbricoides *	2 (1.8)	1 (0.4)	3 (0.8)	—	1 (1.3)	2 (0.7)	—
*Schistosoma mansoni *	2 (1.8)	1 (0.4)	3 (0.8)	—	1 (1.3)	2 (0.7)	
* Enterobius vermicularis *	2 (1.8)	0 (0)	2 (0.5)	—	0 (0)	2 (0.7)	—

**Table 3:** Associating intestinal parasite with CD4 count and diarrheic and ART status among HIV positive study participants at Hiwot Fana Specialized University Hospital, Eastern Ethiopia, 2011.

Variables	No examined (% pos.)	Crude odds ratio (95% confidence interval)	*P* value	Adjusted odds ratio (95% confidence interval)	*P* value
CD_4_ count (cells/*µ*L)					
≥500	75 (30.7)	1		1	
200–499	196 (28.6)	0.90 (0.51, 1.62)	0.734	0.78 (0.42, 1.42)	0.409
<200	100 (46)	1.93 (1.03, 3.61)	0.041	2.07 (1.06, 4.04)	0.034
Diarrheic status					
Nondiarrheic	296 (28.7)	1		1	
Diarrheic	75 (53.3)	2.84 (1.69, 4.77)	0.000	2.30 (1.34, 3.96)	0.003
ART status					
On ART	259 (28.6)	1		1	
Naïve ART	112 (45.5)	2.09 (1.32, 3.31)	0.002	2.60 (1.56, 4.34)	0.000
